# Developmental errors in the common marmoset retina

**DOI:** 10.3389/fnana.2022.1000693

**Published:** 2022-09-20

**Authors:** Silke Haverkamp, Matthias Mietsch, Kevin L. Briggman

**Affiliations:** ^1^Department of Computational Neuroethology, Max Planck Institute for Neurobiology of Behavior – caesar, Bonn, Germany; ^2^Laboratory Animal Science Unit, German Primate Center, Göttingen, Germany; ^3^DZHK (German Center for Cardiovascular Research), Partner Site Göttingen, Göttingen, Germany

**Keywords:** primate retina, misplaced ganglion cell, misplaced bipolar cell, glycinergic amacrine cell, starburst amacrine cell

## Abstract

Although retinal organization is remarkably conserved, morphological anomalies can be found to different extents and varieties across animal species with each presenting unique characteristics and patterns of displaced and misplaced neurons. One of the most widely used non-human primates in research, the common marmoset (*Callithrix jaccus*) could potentially also be of interest for visual research, but is unfortunately not well characterized in this regard. Therefore, the aim of our study was to provide a first time description of structural retinal layering including morphological differences and distinctive features in this species. Retinas from animals (*n* = 26) of both sexes and different ages were immunostained with cell specific antibodies to label a variety of bipolar, amacrine and ganglion cells. Misplaced ganglion cells with somata in the outermost part of the inner nuclear layer and rod bipolar cells with axon terminals projecting into the outer plexiform layer instead of the inner plexiform layer independent of age or sex of the animals were the most obvious findings, whereas misplaced amacrine cells and misplaced cone bipolar axon terminals occurred to a lesser extent. With this first time description of developmental retinal errors over a wide age range, we provide a basic characterization of the retinal system of the common marmosets, which can be taken into account for future studies in this and other animal species. The finding of misplaced ganglion cells and misplaced bipolar cell axon terminals was not reported before and displays an anatomic variation worthwhile for future analyzes of their physiological and functional impact.

## Introduction

Despite enormous differences in cell numbers and cell types between species (Baden et al., 2020; Grünert and Martin, 2020; Shekhar and Sanes, 2021), the basic organization of the retina is remarkably conserved among vertebrates. The major cell classes are arranged in three nuclear layers separated by two synaptic layers. The outer nuclear layer (ONL) is occupied by rod and cone photoreceptor nuclei, the inner nuclear layer (INL) contains horizontal, bipolar and amacrine cells as well as Müller glia cells. The innermost layer, the ganglion cell layer (GCL), contains the retinal ganglion cells (RGCs) whose axons send the visual information to higher visual centers through the optic nerve. Between these three nuclear layers, the two synaptic layers can be found: in the outer plexiform layer (OPL), photoreceptors contact bipolar cells (BCs) and horizontal cells (HCs). In the inner plexiform layer (IPL), BC axon terminals synapse onto amacrine cells (ACs) and RGCs. ACs are presynaptic to BCs, other ACs and RGCs, and receive input from BCs and other ACs, and RGCs are primarily postsynaptic to ACs and BCs (Dowling, 1987). Each coarse cell class (BCs, ACs, RGCs) can be further subdivided into morphologically and functionally distinct cell types that usually form repeating mosaics across the surface of the retina with varying degrees of overlap between neighbors of the same type.

Regardless of this conserved organization, examples of neuronal somata that do not conform to the usual placement described above can be found in all three nuclear layers. These somata have been referred to as displaced cells. An important distinction is whether displaced cells are located outside of the usual nuclear layer but otherwise form a uniform mosaic (i.e., displaced cells that are “intentionally-displaced”) or whether a small proportion of cells of a given cell type is mislocated to a different nuclear layer perhaps due to developmental errors (displaced cells that are truly “misplaced”) (see Vaney, 1990). Misplaced cells often also contain a wiring anomaly in which axons or dendrites stratify in an unusual synaptic layer. The characterization of displaced cells (both intentionally-displaced and misplaced), especially in the context of inter-species comparisons, is of high importance to accurately assess evolutionary adaptations of retinal circuitry. There are numerous examples of displaced retinal cells that are thought to be intentionally-displaced described in a variety of species. Displaced BCs with somata in the ONL are common in turtle (Tauchi, 1990) and tiger salamander retina (Maple et al., 2005), and interestingly, all color-opponent BCs recorded in turtle retina have been displaced cell types (Ammermüller et al., 1995; Haverkamp et al., 1999). Large displaced RGCs with somata in the proximal INL were first described in the bird retina (Dogiel, 1895). These cells exclusively project to the accessory optic nuclei and are responsible for the optokinetic nystagmus in birds, reptiles and amphibians (for review, see Simpson, 1984). In mammals, however, the function of displaced RGCs is unknown. Typically, only about 1 to 2% of the total ganglion cell population is displaced to the INL, but studies of soma diameter and cell morphology suggest that displaced RGCs represent several distinct cell types in mammals and most of them resemble their counterparts in the GCL (Coleman et al., 1987; Buhl and Dann, 1988; Pang and Wu, 2011). An exception are some of the melanopsin-expressing RGCs, which mediate non-image forming visual processes. In human retina, about half of the melanopsin-expressing RGCs (or 80% of the M1 cells) are displaced (Nasir-Ahmad et al., 2019), whereas in rodent retina only 5 to 10% of the cells are displaced (Karnas et al., 2013; Nadal-Nicolás et al., 2014).

ACs are the most diverse cell class in the retina (Shekhar and Sanes, 2021), and vary widely in morphology and function (MacNeil and Masland, 1998; Diamond, 2017). Most ACs express either GABA or glycine, along with acetylcholine or other neuropeptides, and the main division into GABAergic and glycinergic cells is often correlated with dendritic field size (for review, see Vaney, 1990). Glycinergic ACs are small-field cells, and their dendrites are primarily involved in local interactions between the different sublayers of the IPL. GABAergic ACs are wide-field cells providing lateral interactions across the IPL, and their cell bodies are often displaced toward the GCL (Perry and Walker, 1980; Brandon, 1985; Kao and Sterling, 2006). Some ACs, such as the starburst ACs (SACs) occur as mirror-symmetrical populations of regular and displaced cells and form two functional mosaics. OFF SACs have their cell bodies in the INL and stratify in the outer IPL; ON SACs have their cell bodies in the GCL and their dendrites are restricted to the inner IPL (Vaney et al., 1981). The ON SACs are therefore a clear example of a displaced cell type that is otherwise normally-placed. The same holds true for the glypho-immunoreactive ACs previously described in the macaque retina (Majumdar et al., 2008). Other ACs, such as dopaminergic ACs in the ferret retina form a single retinal mosaic, of which 27% of the cells are located in the GCL, the rest in the INL (Eglen et al., 2003). Similar numbers have been reported for a population of secretagogin-positive ACs in the common marmoset retina, where 24–38% of the cells were localized in the GCL (Weltzien et al., 2015). However, ACs with very low numbers in the GCL have been described rather as misplaced than displaced cells, e.g., Wright and Vaney (1999) described an AC type with 98% of its somata in the INL and 2% displaced to the GCL. These displaced cells seem to be misplaced from the regular array of somata in the INL, but their dendrites stratify into the same layers within the IPL where the regular placed cells stratify.

In the mouse retina, 17 types of displaced ACs have been described (Pérez De Sevilla Müller et al., 2007), and displaced ACs make up 59% of the neurons in the GCL (Jeon et al., 1998), although these numbers do not distinguish intentionally-displaced from misplaced cells. Truly misplaced ACs have been reported in the outermost part of the INL with dendrites stratifying into the OPL; these cells are much less common than intentionally-displaced ACs and probably result from migration errors (Kang et al., 2004; Lee et al., 2006). Misplaced horizontal cells have been reported in different mammalian species (Silveira et al., 1989; Peichl and González-Soriano, 1994). In macaque, 3–5% of the H2 cells were misplaced into the GCL with dendrites stratifying mainly in the IPL but also a few ascending into the OPL (Wässle et al., 2000).

Because all the mentioned animal species display special morphological features, there are also certain characteristics to be expected in the common marmoset (*Callithrix jacchus*), a short-lived non-human primate with increasing importance in a variety of research fields (Tardif et al., 2011; Solomon and Rosa, 2014; Park and Silva, 2019). The aim of this study was therefore to quantify displaced cells recently identified during an aging study on the common marmoset retina (Haverkamp et al., 2022) with a focus on misplaced cells arising from putative developmental errors. We used cell specific antibodies to label several types of bipolar and amacrine cells, and the entire population of ganglion cells, and describe here for the first time in a vertebrate retina misplaced ganglion cells with somata in the outermost part of the INL and misplaced bipolar cell axon terminals projecting into the OPL instead of the IPL.

## Materials and methods

### Tissue collection and preparation

Retinal marmoset tissue used in this study was obtained from 26 common marmosets (15 males, 11 females, 2–15 years old). The animals were sacrificed within a broad aging study at the German Primate Center in Göttingen (Mietsch et al., 2020). All procedures were approved by the local animal welfare committee and by the Lower Saxony State Office for Consumer Protection and Food Safety (reference number 33.19-42502-04-17/2496). Housing conditions were in accordance with the law for animal experiments issued by the German government (Tierschutzgesetz) and complied with the European Union guidelines on the welfare of non-human primates used in Research and the European Union (EU directive 2010/63/EU). The animals were anesthetized intramuscularly with a combination of ketamine (50 mg/kg, Ketamin 10%, WDT), xylazine (10 mg/kg, Xylariem 2%, Ecuphar) and atropine (i.p.,1 mg/kg, Atropinsulfat, Dr. Franz Koehler Chemie GmbH) and killed by an overdose of pentobarbital (150–200 mg/kg) intraperitoneal. The eyes were enucleated, the right eye was immersion fixed in 4 % paraformaldehyde (PFA) for 60 min, and the left eye was used for physiological experiments unrelated to this study. Following fixation, the eyes were stored at 4°C in PBS and 0.02% sodium azide. For immunohistochemistry, the retinas were dissected from the eyecup and retinal pieces of defined eccentricities were used as a whole mount or sectioned vertically (60 μm) with a vibratome (Leica VT 1200 S).

The retinal mouse tissue shown in **Figure 6G** came from a transgenic mouse line we used for a bipolar cell study several years ago (Wässle et al., 2009). The mouse line (genetic background C57BL/6J) expressed GFP under the gustducin promoter (GUS8.4GFP; Huang et al., 2003).

### Immunohistochemistry

Immunohistochemical analyses were performed on flat-mounted and sectioned tissue with the primary antibodies listed in Table 1. We used antibodies against RBPMS to label all RGCs (Rodriguez et al., 2014), against acetylcholine (ChAT) to label cholinergic ACs, against tyrosine hydroxylase (TH) to label dopaminergic ACs, against secretagogin (SCGN) to label SCGN+ ACs, and against neuronal nitric oxide synthase (bNOS) to label NOS+ ACs (Weltzien et al., 2015). Antibodies against GlyT1 were used to label glycinergic ACs (Pow and Hendrickson, 1999), antibodies against protein kinase Cα (PKCα) and CD15 to label specific types of BCs (Chan et al., 2001), and antibodies against Ctbp2 to label ribbon synapses in photoreceptor and BC terminals in marmoset retina (Jusuf et al., 2006).

**Table 1:** Primary antibodies used in this study.

**Antibody**	**Antigen**	**Host**	**Dilution**	**Source, cat#, RRID**
CD15	U-937 histiocytic cell line, purified from tissue culture supernatant or ascites by affinity chromatography	Mouse	1:100	BD Pharmingen, 559045, RRID:AB_397181
ChAT	Purified human placental choline acetyltransferase enzyme	Goat	1:200	Millipore, AB144P, RRID:AB_2079751
Ctbp2	Mouse C-terminal binding protein 2, aa 361–445	Mouse	1:5,000	BD Transduction, 612044, RRID:AB_399431
GlyT1	Aa 614–633 from cloned rat GlyT1	Goat	1:5,000	Millipore, AB1770, RRID:AB_90893
bNOS	Synthetic peptide corresponding to amino acids 251–270 of nitric oxide synthase from rat brain	Rabbit	1:5,000	Sigma, N7155, RRID: AB_26079
PKCα	Protein kinase C, regulatory subunit α; peptide sequence: KVNPQFVHPILQSAV	Rabbit	1:5,000	Sigma, P4334, RRID:AB_477345
RBPMS	KLH-conjugated peptide corresponding to a sequence from the N-terminal region of human RNA binding protein with multiple splicing (RBPMS)	Guinea pig	1:500	Millipore, ABN1376, RRID:AB_2687403
SCGN	Recombinant peptide, corresponding to 276 amino acids of human secretagogin fused to His-tag	Sheep	1:200	BioVendor, RD184120100 RRID:AB_2034060
TH	Recombinant protein corresponding to aa 65 to 255 from human tyrosine hydroxylase	Guinea pig	1:1,000	SySy, 213004, RRID:AB_1279449

aa, amino acids.

Antibodies were diluted in PBS, pH7.4, containing 0.5–1% Triton X-100 and 0.02% sodium azide. Immunohistochemical labeling was performed using the indirect fluorescence method. Cryostat and vibratome sections were incubated overnight in the primary antibodies, followed by incubation (1 h) in the secondary antibodies, which were conjugated to Alexa TM 488 (Invitrogen), Cy3 (Dianova), Cy5 or Alexa TM 647. In double labeling experiments, sections were incubated in a mixture of primary antibodies, followed by a mixture of secondary antibodies. Whole mounts were incubated for 2–4 d in the primary and for 2 h in the secondary antibody solution. The number of animals used for the different experiments and immunostainings is given in Table 2. We did not recognize any differences between sexes or ages.

**Table 2:** Number of animals used for immunostaining (age and sex in brackets).

**RBPMS**	**ChaT**	**GlyT1**	**PKCα**
5 (2f, 8m, 9f, 11m, 15f)	10 (2m, 2f, 4f, 6f, 8m, 8f, 9f, 11m, 12m, 14f)	5 (4m, 9m, 9f, 11m, 13m)	17 (2m, 4m, 4m, 5m, 7m, 8m, 8f, 9m, 9f, 11m, 12m, 12m, 12m, 13m, 14f, 14f, 15f)
**CD15**	**bNOS**	**SCGN**	**TH**
5 (2m, 8m, 12f, 13m, 14w)	4 (2f, 4m, 9m, 11m)	4 (2f, 4m, 9m, 11m)	4 (2f, 4m, 9m, 11m)

Ages given in years; m, male; f, female.

### Image acquisition and analysis

Following immunolabeling, retinal tissue samples were mounted in Aqua-Poly/Mount and imaged using confocal microscopy (Leica TCS SP8). Samples were scanned with HC PL APO 20x/0.70 or HC PL APO 40 × /1.3 oil immersion objectives. Voxel size was adjusted with respect to the experimental question. For cell counting, we used the Cell Counter plugin of Image J. Unless stated otherwise, projections of confocal stacks are shown. Images were adjusted in brightness and contrast and occasionally filtered for presentation purposes.

## Results

During screening/characterization of the common marmoset's retinal system in a larger animal cohort of different ages and both sexes, we encountered various unusual cell patterns, which are described in more detail below. Misplaced ganglion cells and starburst amacrine cells in the outer INL as well as misplaced bipolar cell axon terminals in the OPL were the most striking findings.

### Misplaced ganglion cells and starburst amacrine cells in the outer INL

**Figure 1:**
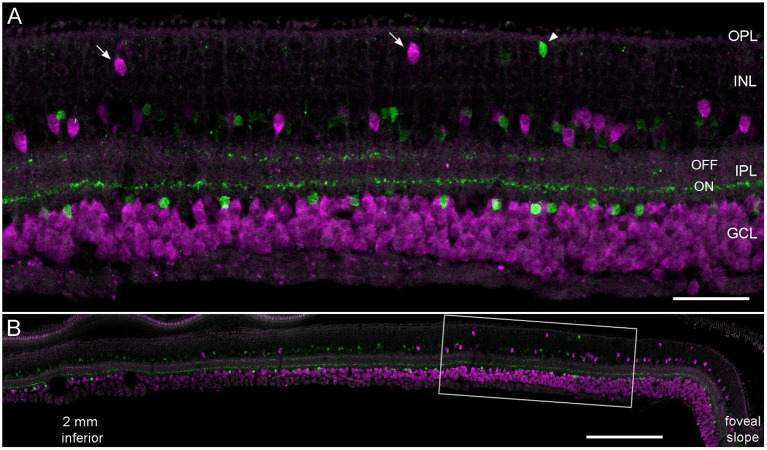
Displaced retinal ganglion cells in the central marmoset retina. Vertical vibratome section of an 11-year-old male double labeled with antibodies against RBPMS (magenta) and ChAT (green). **(A)** Confocal image of the central retina at about 0.8 mm inferior. Arrows mark displaced magenta RGCs; the arrowhead shows a misplaced SAC. **(B)** Same section at lower magnification showing the retina from 2.2 mm inferior to the foveal slope. Scale bar 50 μm for **(A)** and 200 μm for **(B)**.

While estimating the density of RGCs in several adult and aged marmosets (Haverkamp et al., 2022), we realized that displaced RGCs in the inner INL were highly concentrated in the central retina and that even some of them were displaced to the outer INL. The vibratome section in Figure 1A, immunolabeled for a general RGC marker (RBPMS) and ChAT, highlights two outer INL displaced RGCs (arrows) and a putative misplaced starburst amacrine cell (SAC, arrowhead). SACs usually appear as mirror symmetrical populations with OFF SACs stratifying in the outer IPL and ON SACs stratifying in the inner IPL. RGCs in the GCL form one row of somata at 2 mm, 2–3 at 1 mm and up to five rows close to the foveal slope (Figure 1B). Accordingly, many displaced RGCs with somata in the inner INL appear close to the foveal slope and the number decreases significantly already between 1 and 2 mm of eccentricity.

**Figure 2:**
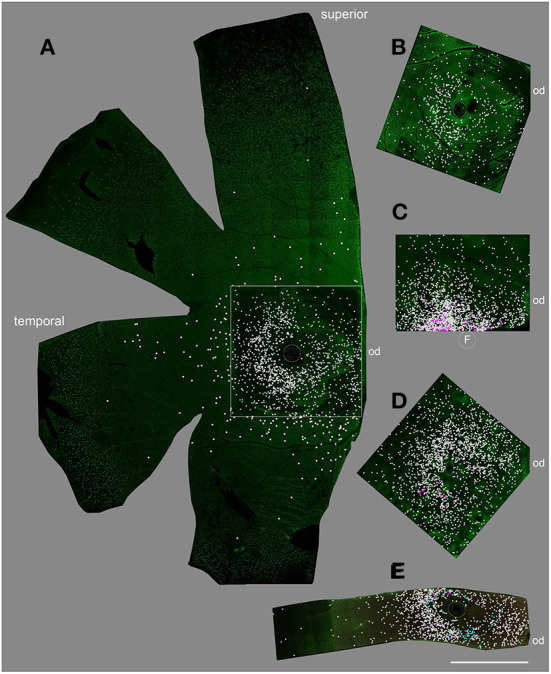
Displaced RGCs in the central retina of five individuals. **(A)** female 15 years old, **(B)** male, 8 years, **(C)** male, 11 years, **(D)** female, 2 years, **(E)** female, 9 years. Flat-mount pieces in **(A–D)** were labeled with RBPMS, in **(E)** double labeled with RBPMS and Satb1. White dots mark the position of inner INL displaced RGCs; magenta dots mark outer INL displaced RGCs and cyan dots in **(E)** mark Satb1+ RGCs. F, fovea; od, optic disc. Scale bar for **(A–E)** 2 mm.

Displaced RGCs near the inner INL boarder were highly concentrated in the central retina of all whole mounts we analyzed (*n* = 5); however, the numbers between individuals varied widely (Figure 2, white dots). The numbers varied from 500 cells to more than 3,000 estimated cells in the central 12 mm^2^ of five retinas (Table 3). The same held true for displaced RGCs in the outer INL: the numbers ranged from zero or just two cells to more than 100 cells per central retina. The large variability between animals suggests that displaced RGCs do not comprise an intentionally-displaced subpopulation but rather are misplaced cells (except for the M1 cells; see introduction). However, due to the high density of RGCs in the central 12 mm^2^ of the GCL (about 700,000 RGCs, see Masri et al., 2019), only 0.1 to 0.5% of the RGCs are misplaced in the INL.

**Table 3:** Number of displaced RGCs in central marmoset retina.

	**Quantified area**	**Inner INL displaced RGCs**	**Outer INL displaced RGCs**
A: Female, 15y	12 mm^2^	1,368	0
B: Male, 8y	12 mm^2^	514	2
C: Male, 11y	6 mm^2^	1,635	57
	*12 mm^2^	~3,270	~114
D: Female, 2y	12 mm^2^	1,807	15
E: Female, 9y	6 mm^2^	1,270	11
	*12 mm^2^	~2,540	~22

Animal ages are displayed in years (Y).

*calculated numbers based on counted numbers for 6 mm^2^.

We stained one retinal piece with RBPMS and antibodies against the transcription factor special AT-rich binding protein 1 (Satb1), a protein expressed by some wide-field RGCs in marmoset retina (Nasir-Ahmad et al., 2022). Interestingly, 61 of the 1,270 inner INL displaced RGCs were Satb1 positive (4.8%; cyan dots in Figure 2E); however, none of the 11 outer INL displaced RGCs was Satb1 positive.

**Figure 3:**
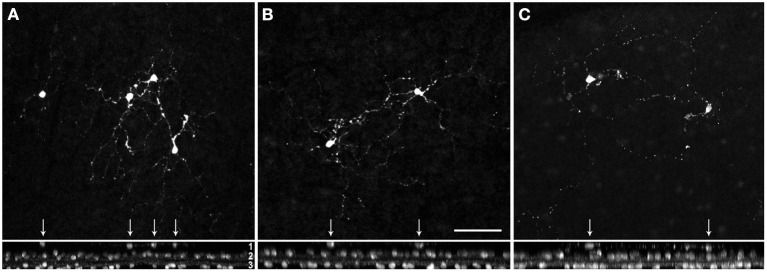
Misplaced cholinergic amacrine cells in the marmoset retina. Examples of misplaced cholinergic ACs in peripheral retina of a 5 year-old female **(A,B)**, and 9 year-old female **(C)**. Z-projections of confocal image stacks are shown in the upper panel, the corresponding xy-projections in the lower panel. Misplaced cells are marked with arrows. Layer 1, somata of the misplaced cells in the outer part of the INL; layer 2, somata of the cholinergic cells in the inner INL (OFF SACs); layer 3, labeled somata in the GCL (ON SACs). Scale in **(B)** 50 μm for **(A–C)**.

Next, we focused on GABAergic ACs and used common markers (TH, NOS, SCGN, ChAT) to label several subpopulations. However, only a few cholinergic cells (with starburst-like morphology) appeared truly misplaced to the outer INL (Figure 3); all other labeled cells (TH, NOS, SCGN) appeared normal, including displaced NOS+ and SCGN+ ACs in the GCL (not shown). We found examples of misplaced ChAT+ starburst-like ACs in 10 animals (Table 2), mainly in the peripheral retina. The somata were located in the outermost part of the INL (layer 1 in Figure 3), with their dendrites extending toward the outer plexiform layer (see also Figure 1A), indicative of a wiring error. The cells appeared much simpler in terms of numbers of branches and branch points compared to ON ChAT cells injected with DiI in marmoset retina (Chandra et al., 2019) and probably comparable to human OFF SACs that appear to have a simpler dendritic branching pattern than ON SACs (Kolb et al., 1992).

### Misplaced glycinergic amacrine cells in the outer INL or OPL

**Figure 4:**
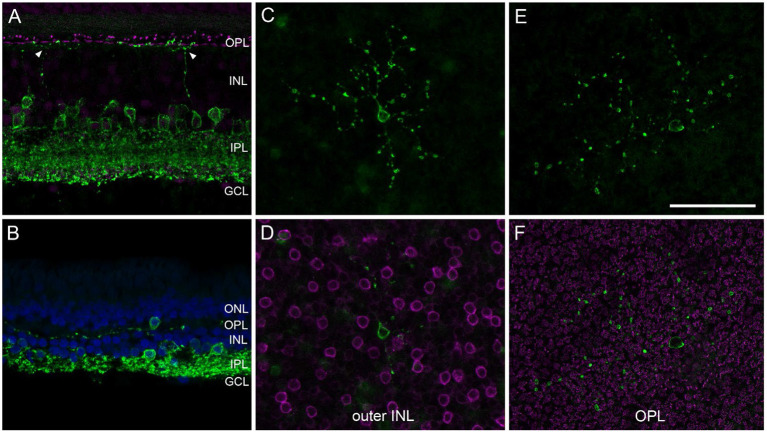
Misplaced glycinergic amacrine cells in the marmoset retina. Vibratome sections or whole mount pieces were immunolabeled with GlyT1 (green) and Ctbp2 [magenta in **(A,F)**] or PKCα [magenta in **(D)**], and counterstained with DAPI [blue in **(B)**]. **(A)** Central vibratome section with two glycinergic cells sending dendrites into the OPL. **(B)** Far peripheral vibratome section with a misplaced glycinergic AC (mGlyAC) within the OPL. **(C–F)** Two examples of mGlyACs with their cell bodies in the outer INL next to PKCα-labeled RBC bodies **(C,D)** and in the OPL in between Ctbp2-labeled ribbon synapses of photoreceptor terminals **(E,F)**, respectively. Z-projections of confocal image stacks are shown in **(C,E)**, confocal images of single sections in **(D,F)**. Scale bar in **(E)** 50 μm for **(A–F)**.

AII ACs are the best-characterized glycinergic ACs in the mammalian retina (for review see Wässle et al., 1995). They present a distinctive type of narrow-field bistratified AC and are crucial interneurons of the rod pathway (Kolb and Famiglietti, 1974). Misplaced AII ACs have so far only been described in the mouse retina (Park et al., 2004; Lee et al., 2006). To our knowledge, there are no reports about other misplaced glycinergic ACs (mGlyACs); recently, however, a new type of interneuron has been described in mouse and primate retina. The so-called Campana cells share some features with BCs, such as receiving input from photoreceptors and relaying visual signals to RGCs, but also share some features with AII ACs, such as their dendritic morphology in the IPL and the expression of GlyT1 (Young et al., 2021). By reproducing their results with our marmoset tissue (*n* = 5, Table 2), we not only saw glycinergic ACs with ascending dendrites into the OPL (Figure 4A, arrowheads), but also readily found mGlyACs with somata in the OPL (Figures 4B,F) or outer INL (Figure 4D).

We imaged several mGlyACs in midperipheral and peripheral retina and recognized different morphological types. Two examples are shown in Figures 4C–F. Some appeared like misplaced AII cells (Figure 4C); others were more asymmetrical and had larger dendritic trees (Figures 4B,E) and two were very small and “knotty” like (not shown, Mariani, 1990).

### Misplaced bipolar cell axon terminals are quite common in marmoset retina

**Figure 5:**
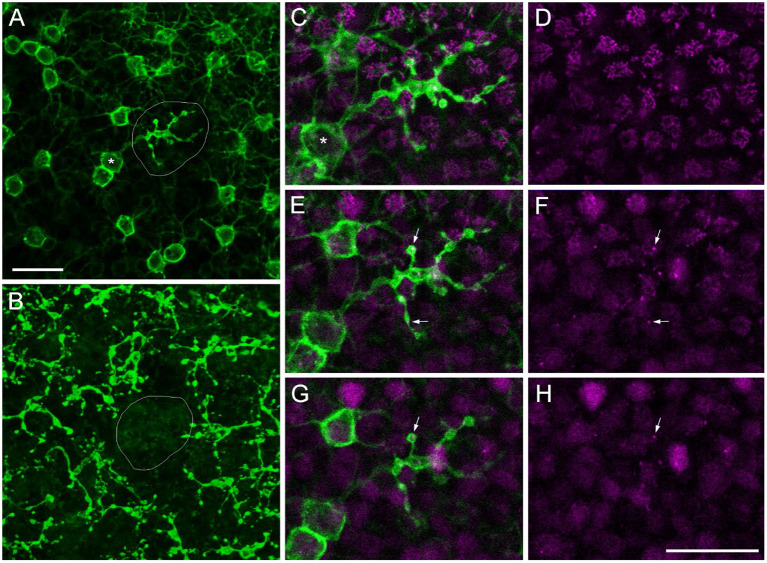
Misplaced rod bipolar axon terminal projecting into the OPL. Confocal image stack of a marmoset retina immunolabeled with antibodies against PKCα (green) and Ctbp2 (magenta). **(A)** Confocal plane at the border of the outer INL to OPL with a misplaced RBC axon terminal (AT) in the middle of the image (circled) surrounded by several other RBC somata and their dendrites projecting into the OPL (upper right). The soma corresponding to the misplaced AT is highlighted (asterisk). **(B)** Same RBCs as in **(A)** with the focus onto their ATs deep in the IPL. The “original” position of the misplaced AT is empty. The misplaced AT is shown in higher magnification in three subsequent confocal planes in **(C,D)** (first plane), **(E,F)** (second plane), and **(G,H)** (third plane). **(C,E,G)** show the PKCα and the Ctpb2 labeling; **(D,F,H)** show Ctbp2 labeling alone. The small arrows point to Ctbp2 puncta within the misplaced AT. Scale bars in **(A)** for **(A,B)** and in **(H)** for **(C–H)** = 20 μm.

During our search for sprouting rod bipolar cells (RBCs) into the ONL (Haverkamp et al., 2022), we were surprised to find RBCs with axon terminals (ATs) projecting into the OPL instead of the IPL in all retinas we stained for PKCα, a rod bipolar cell marker (*n* = 17, Table 2). We often saw brightly labeled ATs in the focal plane of PKCα-labeled somata in the INL and their dendrites reaching into the OPL (Figure 5A), with a missing terminal in the “original” position in the IPL (Figure 5B). Double labeling with Ctbp2 showed that ribbon synapses are also expressed in the misplaced ATs (small arrows in Figures 5C–H), as they usually are in the normal placed ATs in the IPL (see Figure 4 in Neumann and Haverkamp, 2013).

**Figure 6:**
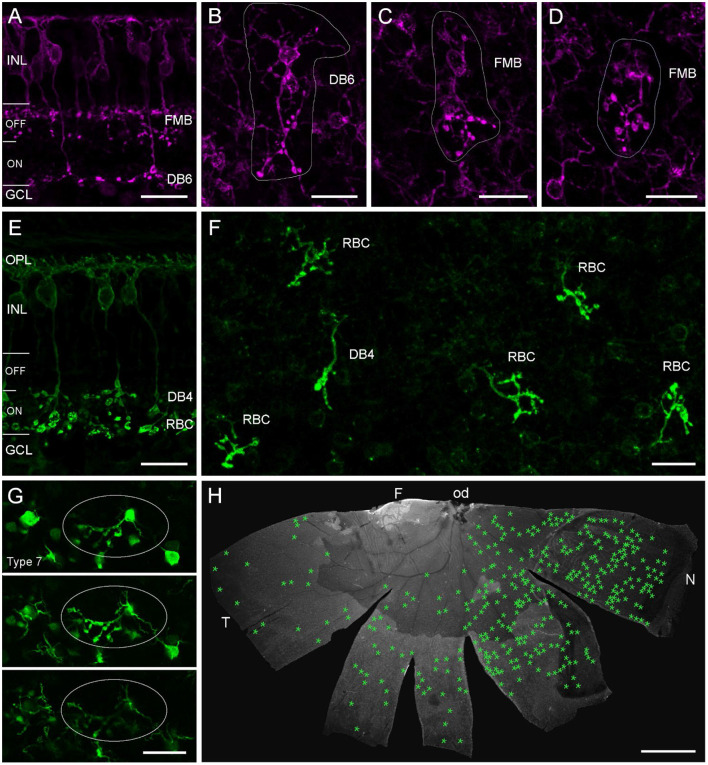
Misplaced bipolar cell axon terminals in marmoset and mouse retina. Confocal images of marmoset retina **(A–F)** and mouse retina from a transgenic line **(G)** immunolabeled for CD15 **(A–D)**, PKCα **(E,F)**, or GFP **(G)**. **(A)** Vertical vibratome section with two CD15+ BC types. The flat midget bipolar (FMB) stratifies in the OFF layer of the IPL and the DB6 in the deep ON layer. **(B)** Example of a DB6 cell with its misplaced AT in the OPL in a whole mount preparation of a 2-year-old male. Soma with dendrites and misplaced AT are encircled. **(C,D)** Examples of two FMBs with misplaced ATs in the same retina as in **(B)**. **(E)** Vertical vibratome section with two PKCα-labeled BC types. Both cell types stratify in the ON layer, the DB4 in S3 of the IPL and the RBC in S4/5. **(F)** Confocal image of several misplaced RBC ATs and one misplaced DB4 AT from the peripheral nasal retina of a 13-year-old male. **(G)** Three consecutive confocal planes of an image stack with GFP-expressing type 7 mouse BCs at the level of the outer INL and OPL. One BC has a misplaced AT projecting into the OPL (circled), soma and dendrites appear at the same plane in their normal position. **(H)** Half retina of the 13-year-old marmoset shown in **(F)**. Green asterisks mark the position of all misplaced ATs (*n* = 349) found in this whole mount preparation. INL, inner nuclear layer; GCL, ganglion cell layer; OPL, outer plexiform layer; F, fovea; od, optic disc; T, temporal; N, nasal. Scale bars, 20 μm in **(A–G)**, 2 mm in **(H)**.

We wondered if we would also find cone bipolar cells (CBCs) with misplaced ATs in the marmoset retina. Therefore, we used CD15 as a marker for flat midget and DB6 BCs (Figure 6A) and labeled retinal whole mount pieces (*n* = 5, Table 2) with CD15 and Ctbp2. Intense screening revealed a few examples of misplaced DB6 and FMB ATs in the outer retina (Figures 6B–D). We also found a few examples of PKCα-labeled misplaced DB4 ATs, which are clearly distinguishable from RBC ATs (Figure 6F) and normally stratify in sublamina 3 of the IPL (Figure 6E) (Boycott and Wässle, 1991; Chan et al., 2001). Although we did not quantify, it was evident that the number of misplaced RBC ATs was much higher than the number of misplaced CBC ATs, at least the sub-types we stained. The number of misplaced RBC ATs ranged from almost 50 to more than 500 per retina. The half retina shown in Figure 6H had a higher concentration on the nasal side.

Finally, we also show an example of a misplaced AT (Figure 6G) from a retinal whole mount of a transgenic mouse line (GUS8.4GFP) previously used to quantify BC types in the mouse retina (Wässle et al., 2009), in which Type 7 BCs and their axons terminating in sublamina 4 of the IPL are prominently labeled (Huang et al., 2003).

## Discussion

As the interest in the common marmoset is continuously increasing, especially in the area of neurobiological research (Solomon and Rosa, 2014), and since the introduction of genetically modified animals will broaden the use of this small non-human primate even further (Park and Silva, 2019), a comprehensive overview of the retina as well as an understanding of this species' morphological variations is necessary. Utilizing a sparse labeling approach, Masri et al. (2019) reported on the variability of ganglion cell morphology in a limited number of marmoset retinas and identified over 17 RGC types, a diversity comparable to other primate retinas (Shekhar and Sanes, 2021), and morphology and stratification resembling RGCs in macaque (Dacey, 2004). Weltzien et al. (2015) reported on cell densities in the common marmoset retina, but the analyses were performed just in the INL in a limited number of animals. Thus, although providing a comprehensive overview over RGC populations and interneuron densities, unfortunately no morphological variations were reported in these studies.

Overall, we did not observe a clear indication of an intentionally-displaced population of RGCs such as the large-displaced RGCs in the bird retina. Given the inter-animal variability we observed, we therefore consider the numerous displaced RGCs preferentially located near the fovea to be misplaced somata and perhaps a reflection of packing density constraints. A similar concentration of displaced RGCs near the visual streak has been documented in the ground squirrel retina (Xiao et al., 2021). We confirmed the intentionally-displaced population of ON SACs, but noted misplaced starburst-like ACs in the outer INL in several retinas with a very unusual extension of their dendrites into the OPL. We are not aware of a report of such misplaced SACs in other vertebrate species except for the mouse retina (Kang et al., 2004).

The misplaced cells we did find ranged from rare occurrences of GABAergic ACs and cone BCs to apparently more common instances of glycinergic ACs and RBCs. Ultimately, the functional significance of misplaced cells, if any, can only be determined by mapping the synaptic connections they form in the retina. This will likely require volumetric electron microscopy data of sufficient spatial extent to capture potentially rare misplaced cells. Many misplaced somata will presumably form typical synaptic connections as long as their axons or dendrites stratify in the typical plexiform layers. Of particular future interest will be examining the synaptic connectivity of misplaced cells with obvious abnormal axonal/dendritic morphologies such as those we observed for several AC and BC types.

In conclusion, we report here for the first time on species-specific/unique cell patterns and distributions of various misplaced and displaced cell populations in the common marmoset's retina. This characterization will not only broaden the knowledge about an increasingly popular non-human primate but will also serve as basic/reference values for future investigations into the visual system of this animal species.

## Data availability statement

The original contributions presented in the study are included in the article/supplementary material, further inquiries can be directed to the corresponding author.

## Ethics statement

The animal study was reviewed and approved by the institutional animal welfare committee of the German Primate Center in Göttingen and by the Lower Saxony State Office for Consumer Protection and Food Safety.

## Author contributions

SH designed the project, analyzed data, and prepared the figures. SH and MM performed experiments. SH, MM, and KB wrote the manuscript. All authors contributed to the article and approved the submitted version.

## Funding

This work was supported by the Center of Advanced European Studies and Research, and by the Max Planck Society.

## Conflict of interest

The authors declare that the research was conducted in the absence of any commercial or financial relationships that could be construed as a potential conflict of interest.

## Publisher's note

All claims expressed in this article are solely those of the authors and do not necessarily represent those of their affiliated organizations, or those of the publisher, the editors and the reviewers. Any product that may be evaluated in this article, or claim that may be made by its manufacturer, is not guaranteed or endorsed by the publisher.

## References

[B1] Ammermüller J. Muller J. F. Kolb H. (1995). The organization of the turtle inner retina. II. Analysis of color-coded and directionally selective cells. J. Comp. Neurol. 358, 35–62. 10.1002/cne.903580103 7560276

[B2] Baden T. Euler T. Berens P. (2020). Understanding the retinal basis of vision across species. Nat. Rev. Neurosci. 21, 5–20 10.1038/s41583-019-0242-1 31780820

[B3] Boycott B. B. Wässle H. (1991). Morphological classification of bipolar cells of the primate retina. Eur. J. Neurosci. 3, 1069–1088. 10.1111/j.1460-9568.1991.tb00043.x 12106238

[B4] Brandon C. (1985). Retinal GABA neurons: localization in vertebrate species using an antiserum to rabbit brain glutamate decarboxylase. Brain Res. 344, 286–295. 10.1016/0006-8993(85)90806-6 2994837

[B5] Buhl E. H. and Dann, J. F. (1988). Morphological diversity of displaced retinal ganglion cells in the rat: a lucifer yellow study. J. Comp. Neurol. 269, 210–218 10.1002/cne.902690206 3356810

[B6] Chan T. L. Martin P. R. Clunas N. Grünert U. (2001). Bipolar cell diversity in the primate retina: morphologic and immunocytochemical analysis of a new world monkey, the marmoset Callithrix jacchus. J. Comp. Neurol. 437, 219–239. 10.1002/cne.1280 11494253

[B7] Chandra A. J. Lee S. C. S. Grünert U. (2019). Melanopsin and calbindin immunoreactivity in the inner retina of humans and marmosets. Vis. Neurosci. 36, E009. 10.1017/S0952523819000087 31581958

[B8] Coleman L. A. Harman A. M. Beazley L. D. (1987). Displaced retinal ganglion cells in the wallaby Setonix brachyurus. Vision Res. 27, 1269–1277. 10.1016/0042-6989(87)90203-3 3424674

[B9] Dacey D. M. (2004). “Origins of perception: retinal ganglion cell diversity and the creation of parallel visual pathways,“ in *The Cognitive Neurosciences, 3rd Edn*, ed M. S. Gazzaniga (Cambridge: MIT Press), 281–301.

[B10] Diamond J. S. (2017). Inhibitory interneurons in the retina: types, circuitry, and function. Annu. Rev. Vis. Sci. 3, 1–24. 10.1146/annurev-vision-102016-061345 28617659

[B11] Dogiel A. S. (1895). Die retina der vögel. Anat Entwicklungsgeschichte 44, 622–648. 10.1007/BF02934032

[B12] Dowling J. E. (1987). The Retina: An Approachable Part of the Brain. Cambridge, Mass: Belknap Press of Harvard University Press.

[B13] Eglen S. J. Raven M. A. Tamrazian E. Reese B. E. (2003). Dopaminergic amacrine cells in the inner nuclear layer and ganglion cell layer comprise a single functional retinal mosaic. J. Comp. Neurol. 466, 343–355 10.1002/cne.10891 14556292

[B14] Grünert U. and Martin, P. R. (2020). Cell types and cell circuits in human and non-human primate retina. Prog. Retin. Eye Res. 78, 100844. 10.1016/j.preteyeres.2020.100844 32032773

[B15] Haverkamp S. Möckel W. Ammermüller J. (1999). Different types of synapses with different spectral types of cones underlie color opponency in a bipolar cell of the turtle retina. Vis. Neurosci. 16, 801–809. 10.1017/S0952523899164186 10580716

[B16] Haverkamp S. Reinhard K. Peichl L. Mietsch M. M. (2022). No evidence for age-related alterations in the marmoset retina. Front. Neuroanat. 16:945295. 10.3389/fnana.2022.945295 36120100 PMC9479465

[B17] Huang L. Max M. Margolskee R. F. Su H. Masland R. H. Euler T. (2003). G protein subunit Gγ13 is coexpressed with Gαo, Gβ3, and Gβ4 in retinal ON bipolar cells. J. Comp. Neurol. 455, 1–10. 10.1002/cne.10396 12454992

[B18] Jeon C. J. Strettoi E. Masland R. H. (1998). The major cell populations of the mouse retina. J. Neurosci. 18, 8936–8946. 10.1523/JNEUROSCI.18-21-08936.1998 9786999 PMC6793518

[B19] Jusuf P. R. Martin P. R. Grünert U. (2006). Synaptic connectivity in the midget-parvocellular pathway of primate central retina. J. Comp. Neurol. 494, 260–274. 10.1002/cne.20804 16320234

[B20] Kang T. H. Ryu Y. H. Kim I. B. Oh G. T. Chun M. H. (2004). Comparative study of cholinergic cells in retinas of various mouse strains. Cell Tissue Res. 317, 109–115. 10.1007/s00441-004-0907-5 15221444

[B21] Kao Y.-H. and Sterling, P. (2006). Displaced GAD65 amacrine cells of the guinea pig retina are morphologically diverse. Vis. Neurosci. 23, 931–939. 10.1017/S0952523806230293 17266785

[B22] Karnas D. Mordel J. Bonnet D. Pévet P. Hicks D. Meissl H. (2013). Heterogeneity of intrinsically photosensitive retinal ganglion cells in the mouse revealed by molecular phenotyping. J. Comp. Neurol. 521, 912–932. 10.1002/cne.23210 22886938

[B23] Kolb H. and Famiglietti, E. V. (1974). Rod and cone pathways in the inner plexiform layer of cat retina. Science 186, 4749. 10.1126/science.186.4158.47 4417736

[B24] Kolb H. Linberg K. A. Fisher S. K. (1992). Neurons of the human retina: a Golgi study. J. Comp. Neurol. 318, 147–187. 10.1002/cne.903180204 1374766

[B25] Lee E. J. Mann L. B. Rickman D. W. Lim E. J. Chun M. H. Grzywacz N. M. (2006). AII amacrine cells in the distal inner nuclear layer of the mouse retina. J. Comp. Neurol. 494, 651–62. 10.1002/cne.20838 16374803

[B26] MacNeil M. A. and Masland, R. H. (1998). Extreme diversity among amacrine cells: implications for function. Neuron 20, 971–982. 10.1016/S0896-6273(00)80478-X 9620701

[B27] Majumdar S. Wässle H. Jusuf P. R. Haverkamp S. (2008). Mirror-symmetrical populations of wide-field amacrine cells of the macaque monkey retina. J. Comp. Neurol. 508, 13–27. 10.1002/cne.21666 18288700

[B28] Maple B. R. Zhang J. Pang J. J. Gao F. Wu S. M. (2005). Characterization of displaced bipolar cells in the tiger salamander retina. Vision Res. 45, 697–705. 10.1016/j.visres.2004.09.038 15639496

[B29] Mariani A. P. (1990). Amacrine cells of the rhesus monkey retina. J. Comp. Neurol. 301, 382–400. 10.1002/cne.903010305 2262597

[B30] Masri R. A. Percival K. A. Koizumi A. Martin P. R. Grünert U. (2019). Survey of retinal ganglion cell morphology in marmoset. J. Comp. Neurol. 527, 236–258. 10.1002/cne.24157 27997691

[B31] Mietsch M. Paqué K Drummer C. Stahl-Hennig C. Roshani B. (2020). The aging common marmoset's immune system: from junior to senior. Am. J. Primatol. 82, e23128. 10.1002/ajp.23128 32246726

[B32] Nadal-Nicolás F. M. Salinas-Navarro M. Jiménez-López M. Sobrado-Calvo P. Villegas-Pérez M. P. Vidal-Sanz M.. (2014). Displaced retinal ganglion cells in albino and pigmented rats. Front. Neuroanat. 8, 99. 10.3389/fnana.2014.00099 25339868 PMC4186482

[B33] Nasir-Ahmad S. Lee S. C. S. Martin P. R. Grünert U. (2019). Melanopsin-expressing ganglion cells in human retina: morphology, distribution, and synaptic connections. J. Comp. Neurol. 527, 312–327. 10.1002/cne.24176 28097654

[B34] Nasir-Ahmad S. Vanstone K. A. Novelli M. Lee S. C. S. Do M. T. H. Martin P. R.. (2022). Satb1 expression in retinal ganglion cells of marmosets, macaques, and humans. J. Comp. Neurol. 530, 923–940. 10.1002/cne.25258 34622958 PMC8831458

[B35] Neumann S. and Haverkamp, S. (2013). Characterization of small-field bistratified amacrine cells in macaque retina labeled by antibodies against synaptotagmin-2. J. Comp. Neurol. 521, 709–724. 10.1002/cne.23201 22821706

[B36] Pang J. J. and Wu, S. M. (2011). Morphology and immunoreactivity of retrogradely double-labeled ganglion cells in the mouse retina. Invest. Ophthalmol. Vis. Sci. 52, 4886–4896. 10.1167/iovs.10-5921 21482641 PMC3175970

[B37] Park J. E. and Silva, A. C. (2019). Generation of genetically engineered non-human primate models of brain function and neurological disorders. Am. J. Primatol. 81, e22931. 10.1002/ajp.22931 30585654 PMC6463491

[B38] Park S. J. Lim E. J. Oh S. J. Chung J. W. Rickman D. W. Moon J. I.. (2004). Ectopic localization of putative AII amacrine cells in the outer plexiform layer of the developing FVB/N mouse retina. Cell Tissue Res. 315, 407–412. 10.1007/s00441-003-0844-8 14722751

[B39] Peichl L. and González-Soriano, J. (1994). Morphological types of horizontal cell in rodent retinae: a comparison of rat, mouse, gerbil, and guinea pig. Vis. Neurosci. 11, 501–517. 10.1017/S095252380000242X 8038125

[B40] Pérez De Sevilla Müller L. Shelley J. Weiler R. (2007). Displaced amacrine cells of the mouse retina. J. Comp. Neurol. 505, 177–189. 10.1002/cne.21487 17853452

[B41] Perry V. H. and Walker, M. (1980). Amacrine cells, displaced amacrine cells and interplexiform cells in the retina of the rat. Proc. R. Soc. Lond. B. Biol. Sci. 208, 415–431. 10.1098/rspb.1980.0060 6158054

[B42] Pow D. V. and Hendrickson, A. E. (1999). Distribution of the glycine transporter glyt-1 in mammalian and nonmammalian retinae. Visual Neurosci. 16, 231–239. 10.1017/S0952523899162047 10367958

[B43] Rodriguez A. R. de Sevilla Müller L. P. Brecha N. C. (2014). The RNA binding protein RBPMS is a selective marker of ganglion cells in the mammalian retina. J. Comp. Neurol. 522, 1411–1443. 10.1002/cne.23521 24318667 PMC3959221

[B44] Shekhar K. and Sanes, J. R. (2021). Generating and using transcriptomically based retinal cell atlases. Annu. Rev. Vis. Sci. 7, 43–72. 10.1146/annurev-vision-032621-075200 34228938

[B45] Silveira L. C. Yamada E. S. Picanco-Diniz C. W. (1989). Displaced horizontal cells and biplexiform horizontal cells in the mammalian retina. Vis. Neurosci. 3, 483–488. 10.1017/S0952523800005988 2487119

[B46] Simpson J. I. (1984). The accessory optic system. Annu. Rev. Neurosci. 7, 13–41. 10.1146/annurev.ne.07.030184.000305 6370078

[B47] Solomon S. G. and Rosa, M. G. (2014). A simpler primate brain: the visual system of the marmoset monkey. Front. Neural Circuits 8, 96. 10.3389/fncir.2014.00096 25152716 PMC4126041

[B48] Tardif S. D. Mansfield K. G. Ratnam R. Ross C. N. Ziegler T. E. (2011). The marmoset as a model of aging and age-related diseases. ILAR journal / National Research Council, Institute of Laboratory Animal Resources 52, 54–65. 10.1093/ilar.52.1.54 21411858 PMC3775658

[B49] Tauchi M. (1990). Single cell shape and population densities of indoleamine-accumulating and displaced bipolar cells in Reeves' turtle retina. Proc. R. Soc. Lond. B. Biol. Sci. 238, 351–367. 10.1098/rspb.1990.0004 1968643

[B50] Vaney D. I. (1990). The mosaic of amacrine cells in the mammalian retina. Prog. Ret. Res. 9, 49–100. 10.1016/0278-4327(90)90004-2

[B51] Vaney D. I. Peichl L. Boycott B. B. (1981). Matching populations of amacrine cells in the inner nuclear and ganglion cell layers of the rabbit retina. J. Comp. Neurol. 199, 373–391. 10.1002/cne.901990305 6114966

[B52] Wässle H. Dacey D. M. Haun T. Haverkamp S. Grünert U. Boycott B. B. (2000). The mosaic of horizontal cells in the macaque monkey retina: with a comment on biplexiform ganglion cells. Vis. Neurosci. 17, 591–608 10.1017/S0952523800174097 11016578

[B53] Wässle H. Grünert U. Chun M.-H. Boycott B. B. (1995). The rod pathway of the macaque monkey retina: identification of AII-amacrine cells with antibodies against calretinin. J. Comp. Neurol. 361, 537–551. 10.1002/cne.903610315 8550898

[B54] Wässle H. Puller C. Müller F. Haverkamp S. (2009). Cone contacts, mosaics, and territories of bipolar cells in the mouse retina. J. Neurosci. 29, 106–117. 10.1523/JNEUROSCI.4442-08.2009 19129389 PMC6664901

[B55] Weltzien F. Percival K. A. Martin P. R. Grünert U. (2015). Analysis of bipolar and amacrine populations in marmoset retina. J. Comp. Neurol. 523, 313–334. 10.1002/cne.23683 25262625

[B56] Wright L. L. and Vaney, D. I. (1999). The fountain amacrine cells of the rabbit retina. Vis. Neurosci. 16, 1145–1156. 10.1017/S0952523899166148 10614594

[B57] Xiao X. Zhao T. Miyagishima K. J. Chen S. Li W. Nadal-Nicolás F. M. (2021). Establishing the ground squirrel as a superb model for retinal ganglion cell disorders and optic neuropathies. Lab. Invest. 101, 1289–1303. 10.1038/s41374-021-00637-y 34253851 PMC8753557

[B58] Young B. K. Ramakrishnan C. Ganjawala T. Wang P. Deisseroth K. Tian N. (2021). An uncommon neuronal class conveys visual signals from rods and cones to retinal ganglion cells. Proc. Natl. Acad. Sci. USA. 118, e2104884118. 10.1073/pnas.2104884118 34702737 PMC8612366

